# Effect of Oxidative Stress on Cardiovascular System in Response to Gravity

**DOI:** 10.3390/ijms18071426

**Published:** 2017-07-04

**Authors:** Ken Takahashi, Hiroki Okumura, Rui Guo, Keiji Naruse

**Affiliations:** 1 Department of Cardiovascular Physiology, Graduate School of Medicine, Dentistry and Pharmaceutical Sciences, Okayama University, Okayama 700-8558, Japan; kakuei0830@gmail.com (R.G.); knaruse@md.okayama-u.ac.jp (K.N.); 2 Department of Medicine, Okayama University, Okayama 700-8558, Japan; pbuu59vj@s.okayama-u.ac.jp; 3 Department of Cardiovascular Surgery, Harbin Medical University, Harbin 150001, China

**Keywords:** oxidative stress, reactive oxygen species, radiation, microgravity

## Abstract

Long-term habitation in space leads to physiological alterations such as bone loss, muscle atrophy, and cardiovascular deconditioning. Two predominant factors—namely space radiation and microgravity—have a crucial impact on oxidative stress in living organisms. Oxidative stress is also involved in the aging process, and plays important roles in the development of cardiovascular diseases including hypertension, left ventricular hypertrophy, and myocardial infarction. Here, we discuss the effects of space radiation, microgravity, and a combination of these two factors on oxidative stress. Future research may facilitate safer living in space by reducing the adverse effects of oxidative stress.

## 1. Introduction

Five million years after the birth of humankind, we are living in the space age, with the International Space Station continuously accommodating crew members orbiting around the Earth, planning commercial flights to the Moon, and even discussing Mars exploration realistically [1,2,3]. These frontiers excite humanity. We can pursue it and are destined to do so. However, as the duration of stay in space extends to months and years, it has gradually become evident that the space environment affects our physiological functions. A few obvious alterations were identified in the earlier days of space exploration: bone loss [4,5,6], muscle atrophy [5,6,7], and cardiovascular deconditioning, of which orthostatic intolerance is one of the symptoms [6,8,9,10]. Analysis of the long-term effects of spaceflight on human health requires several decades.

The Apollo program was a magnificent project that embodied science technology and exploration, sending 24 astronauts from the Earth to the lunar orbit. While the program represented a significant and unshakable milestone in human history, an alarming fact regarding health risks was reported 40 years later; this report indicated that the Apollo lunar astronauts show higher cardiovascular disease mortality rate [11], caused by heart failure, myocardial infarction, stroke, brain aneurysm, or blood clots than their counterparts who experienced the space environment only at low Earth orbit (LEO) and who did not experience space travel [11]. The authors of this report, which include a researcher from the National Aeronautics and Space Administration (NASA), assumed that the reason for the higher mortality is space radiation, based on an experiment in mice. Meanwhile, a serious opposing opinion on this report was expressed in terms of the method of data collection and analysis [12]. Therefore, adequate care should be taken to consider the cardiovascular disease mortality rate in response to space radiation. However, it is of great importance to scrutinize the possible effect of the space environment on human health.

One of the alterations caused by long-term space stay is the development of pro-oxidative conditions, including elevated expression of oxidative enzymes (e.g., nicotinamide adenine dinucleotide phosphate (NADP^+^) oxidase (NOX)) and decreased expression of anti-oxidative enzymes (e.g., superoxide dismutase, SOD, and glutathione peroxidase, GPx). Pro-oxidative conditions are observed in spaceflight and simulated space environments (radiation and microgravity) in various types of organs and cells, including erythrocytes [13,14], endothelial cells [15], retina [16], skin [17], brain [18,19], neuronal cells [20], liver [21,22], and skeletal muscles [23,24]. In several studies, the direct detection of increased reactive oxygen species (ROS) or the detection of substances produced by oxidative reactions were reported [13,16,18,20,21,23], implying that the production of oxidative substances increases in the space environment. In this review, we first provide an overview of oxidative stress; then, we discuss the generation of oxidative stress in response to radiation, microgravity, and a combination of these two factors.

## 2. What Are Reactive Oxygen Species (ROS)?

ROS are oxidizing agents produced by both endogenous (mitochondria, peroxisomes, lipoxygenases, NOX, and cytochrome P450) and exogenous (ultraviolet light, ionizing radiation, chemotherapeutics, inflammatory cytokines, and environmental toxins) factors [25]. Superoxide anion (O_2_^−^) [26], hydrogen peroxide (H_2_O_2_), and hydroxyl radicals (OH∙) are the major types of ROS—each of which has preferential biological targets based on its chemical properties [27]. ROS have two different actions. First, with their unstable and highly reactive chemical properties, ROS react with lipids, proteins, and DNA [27], resulting in aging, disease, and cell death [25,28]. Second, in contrast to the first destructive action, ROS are involved in cellular homeostatic functions such as proliferation [29,30] via heat-shock transcription factor 1, nuclear factor-κB, p53, phosphoinositide 3-kinase, and mitogen-activated protein kinase pathways [25]. In contrast to the pro-oxidative process and enzymes described above, SOD, peroxiredoxin, glutathione reductase, GPx, and catalase (CAT) are anti-oxidative enzymes that reduce the levels of ROS.

### ROS in the Cardiovascular System

**Figure 1:**
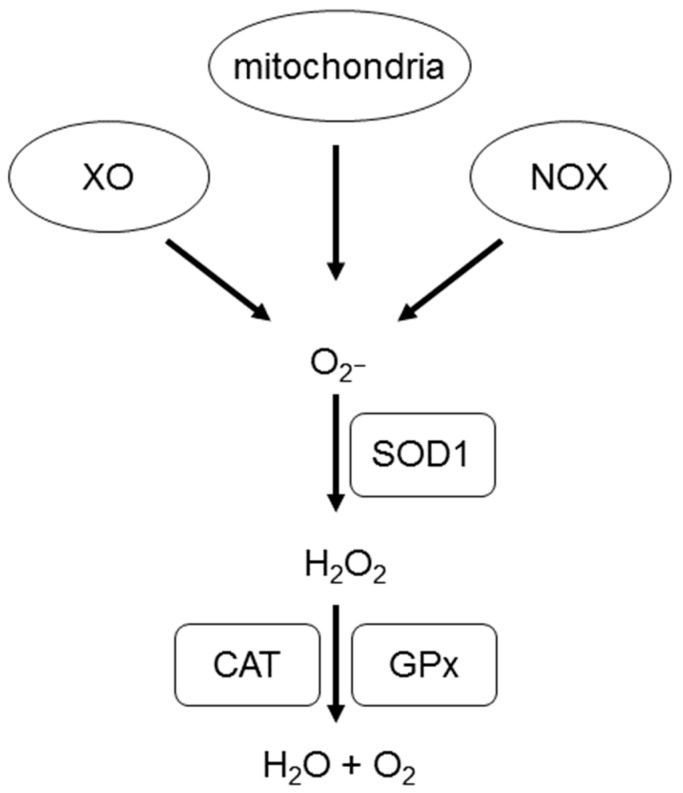
Generation of reactive oxygen species. Superoxide anion is produced by xanthine oxidase (XO), mitochondria, and NADP^+^ oxidase (NOX). Superoxide anion is converted to hydrogen peroxide (H_2_O_2_) by superoxide dismutase 1 (SOD1), and then to H_2_O and O_2_ by catalase (CAT) and glutathione peroxidase (GPx).

As described by Sugamura and Keaney [31], biological processes in the mitochondrial respiratory chain and subsequent enzymatic processes cause ROS generation in the cardiovascular system. The enzymes involved in these processes are NOX, xanthine oxidase (XO), lipoxygenase, nitric oxide synthase (NOS), and myeloperoxidase (Figure 1). Subtypes of NOX proteins are widely expressed in the cardiovascular system—specifically NOX1 (vascular smooth muscle cells), NOX2 (endothelium, vascular smooth muscle cells, adventitia, and cardiomyocytes), NOX4 (endothelium, vascular smooth muscle cells, cardiomyocytes, and cardiac stem cells), and NOX5 (vascular smooth muscle cells) [32]. NOX is involved in the development of cardiovascular diseases such as hypertension, left ventricular hypertrophy, and myocardial infarction [32]. Besides, NOX is also involved in cardiovascular physiology including angiogenesis [33] and blood pressure regulation [34].

Endothelial dysfunction is a hallmark of cardiovascular diseases [35]. An increase in oxidative stress leads to monomerization of the endothelial isoform of NOS (eNOS), which in turn causes further production of superoxide anion rather than nitric oxide [36]. Insufficiency of the nitric oxide production contributes to endothelial dysfunction and the resultant cardiovascular disorders, including hypertension [37].

## 3. ROS Generation in Response to Radiation

Exposure to hazardous radiation from galactic cosmic rays and solar particle events such as solar flares significantly decreases at the altitude of LEO and below due to shielding by the Earth’s atmosphere and magnetosphere [38]. Therefore, the cause of higher cardiovascular risk in the Apollo lunar astronauts is inferred to be severe deep space radiation [11]. Indeed, space radiation causes adverse effects such as DNA damage [39,40] and cell senescence [41]. The adverse effects of radiation are firstly due to direct damage to cellular structures such as DNA, which are exposed to radiation. Secondly, radiation decomposes water molecules to ROS such as O_2_^−^, OH·, and H_2_O_2_ [42]. Thirdly, high-charge and high-energy (HZE) ion particle radiation—which is a component of galactic cosmic rays—generates secondary radiation around its initial location [43]. Lastly, ROS may spread to nearby cells and cause long-term damage [44]. It is reported that long-term space stay affects neuronal function [45,46,47], possibly due to the effects of space radiation on the nervous system [48,49].

### Cardiovascular ROS Generation in Response to Radiation

In the cardiovascular system, radiation causes ischemic heart disease [50], cardiomyopathy, and stroke [51,52]. Supporting this fact is the observation that exposure to HZE radiation facilitates the activation of XO (Figure 1) and a resultant increase of ROS in vascular endothelial cells in rats [53]. Furthermore, XO activity was elevated, and aortic stiffness was higher, even 4 and 6 months after a single radiation exposure, respectively. Increased XO expression in response to HZE radiation was confirmed in mouse endothelial cells as well [11]. In terms of the long-term effects of HZE radiation, elevated ROS and mitochondrial superoxide were observed in intestinal epithelial cells, along with increased NOX expression and decreased SOD and CAT expressions, 1 year after exposure [54].

## 4. ROS Generation in Response to Microgravity

The predominant mechanism of ROS generation in microgravity conditions seems to be the upregulation of oxidative enzymes and downregulation of anti-oxidative enzymes. For example, simulated microgravity has been reported to induce a decrease in the antioxidant enzymes SOD, GPx, and CAT and an increase in the amount of ROS in rat neuronal PC12 cells 96 h after the onset [20]. A similar increase in ROS production was observed in another neuronal cell line, SH-SY5Y [55]. Decreased expression of the anti-oxidative enzyme CAT in the soleus muscle was reported in mice habituated in space for 30 days [56]. Wise et al. reported increased ROS and decreased glutathione levels in response to simulated microgravity using hind limb unloading in the brainstem and frontal cortex of mice [19]. Lipid peroxidation was observed over a wide range of areas in the brain, including the brainstem, cerebellum, frontal cortex, hippocampus, and striatum. In erythrocytes, increased lipid peroxidation was observed after spaceflight [13].

Bed rest in a 6°-head-down tilt posture is often used to simulate the effect of microgravity using human subjects. The unloading condition derived from bedrest induces pro-oxidative conditions, namely decreased expression of the genes related to antioxidation, such as cytochrome c, nicotinamide nucleotide transhydrogenase, and glutathione S-transferase κ1 [57]. Using a hindlimb unloading (HLU) rodent model is another experimental method frequently used to simulate the effect of microgravity. Increased ROS generation along with a decrease in the anti-oxidative protein SOD was observed in rat hippocampus in response to HLU [58].

### Cardiovascular ROS Generation in Response to Microgravity

Another study demonstrated that 3-week HLU caused an increase in superoxide anion levels in the basilar and carotid arteries of rats via the local renin–angiotensin system [59]. In this study, upregulated expression of eNOS was observed in the carotid artery. The effect of microgravity on the cardiovascular system seems to be different depending on the region. Although 4-week hindlimb unloading led to an increase in superoxide levels along with an elevation in the levels of the pro-oxidative enzymes NOX2 and NOX4 and a decrease in the levels of the anti-oxidative enzymes Mn-SOD and GPx-1 in cerebral arteries, this effect was not observed in mesenteric arteries [60,61]. In human umbilical vein endothelial cells, the expression of pro-oxidative thioredoxin-interacting protein was increased in response to 10-day spaceflight [15].

## 5. Combination of Radiation and Microgravity

The effect of a combination of radiation and microgravity on ROS generation seems to be synergistic. Mao et al. studied the effect of low-dose radiation (LDR) and microgravity on oxidative damage in mouse brain, using HLU to simulate microgravity [18]. Surprisingly, exposure to a combination of LDR and HLU—but not LDR or HLU alone—for 7 days caused lipid oxidation in the brain cortex. After 9 months of exposure, lipid peroxidation was observed in the LDR and HLU conditions alone, but was more evident in the LDR + HLU condition. A stronger effect from the combination than from LDR alone was similarly observed in the hippocampus. Therefore, radiation and microgravity have been suggested to have a synergistic effect on lipid oxidation. A synergistic effect was implied in NOX2 expression as well. In contrast, Mao et al. showed reduced SOD activity in simulated microgravity [18]. This effect seems to be specific to the microgravity condition.

ROS production in mouse embryonic stem cells in the presence of H_2_O_2_ that mimics the effect of radiation exposure increased in response to simulated microgravity [62]. The production of ROS from any of the ROS sources (e.g., mitochondria, XO, endothelial NOS, and NOX) can facilitate ROS production from the other sources [63]. This may be the underlying mechanism behind the synergistic effect of radiation and microgravity on the ROS production described above.

## 6. Conclusions

The space environment is predominantly characterized by space radiation and microgravity. These two factors are prominently involved in ROS generation in biological systems. As we discussed here, ROS production is facilitated in specific organs and tissues, including neuronal and cardiovascular systems. However, Stein et al. reported that oxidative stress decreases during spaceflight and then increases after returning to Earth, based on the measurement of a biomarker of lipid oxidation—8-iso-prostaglandin F2α—in urine [64,65]. The discrepancy between organ/tissue/cellular level increase and individual level decrease of oxidative stress during space flight may be attributable to several factors. Firstly, food intake decreases [66,67] and anabolic response is impaired [68] in spaceflight, which may decrease the production of ROS in mitochondria. Secondly, the extent of oxidative stress can be different among tissue types. While several tissues described above show pro-oxidative conditions, some tissue types such as fibroblasts [13] and macrophages [69] show anti-oxidative conditions. Thirdly, the detection sensitivity for oxidative stress from samples such as blood or urine can vary depending on the method. A combination of biomarkers is suggested to provide a more accurate assessment of oxidative stress [70].

In this review, we mainly discussed the alteration of the expression level of ROS-related proteins. However, response to gravity is a mechanobiological process. While the role of the hippo pathway—which is involved in mechanosensitive control of organ size [71]—in gravity sensing has been reported [72,73], much remains to be elucidated to understand the mechanotransduction of gravity.

Men age faster in space. Physiological changes in microgravity and the aging process share common features, such as muscle and bone atrophy, balance and coordination problems (after returning to 1 *g* environment), decreased functional capacity of the cardiovascular system, mild hypothyroidism, increased stress hormones, decreased sex steroids, impaired anabolic response to food intake, and systemic inflammatory response [68,74]. According to NASA researchers Vernikos and Schneider, the processes of muscle and bone atrophy and loss of functional capacity of the cardiovascular system occur about ten times faster in space than on Earth [74]. In contrast, oxidative stress—which we discussed here in the context of a process derived from space radiation and microgravity—is thought to be crucial for the aging process [75]. Understanding oxidative stress in the space environment may help us understand the aging process and contribute to solving the problem of aging. Future research in this field will open the door to a safe passage to Mars and beyond.

## Abbreviations

LEO	Low Earth Orbit
NASA	National Aeronautics and Space Administration
ROS	Reactive Oxygen Species
NADP^+^	Nicotinamide Adenine Dinucleotide Phosphate
NOX	Nicotinamide Adenine Dinucleotide Phosphate Oxidase
XO	Xanthine Oxidase
NOS	Nitric Oxide Synthase
SOD	Superoxide Dismutase
GPx	Glutathione Peroxidase
CAT	Catalase
HZE	High-Charge and High-Energy
LDR	Low-Dose Radiation
HLU	Hindlimb Unloading
eNOS	Endothelial Isoform of Nitric Oxide Synthase

## References

[1] Witze A. (2016). NASA rethinks approach to Mars exploration. Nature.

[2] Cucinotta F.A. (2015). Review of NASA approach to space radiation risk assessments for Mars exploration. Health Phys..

[3] Messina P., Vennemann D. (2005). The European space exploration programme: Current status of ESA’s plans for Moon and Mars exploration. Acta Astronaut..

[4] Grimm D., Grosse J., Wehland M., Mann V., Reseland J.E., Sundaresan A., Corydon T.J. (2016). The impact of microgravity on bone in humans. Bone.

[5] Stein T.P. (2013). Weight, muscle and bone loss during space flight: Another perspective. Eur. J. Appl. Physiol..

[6] Bullard R.W. (1972). Physiological problems of space travel. Annu. Rev. Physiol..

[7] Narici M.V., de Boer M.D. (2011). Disuse of the musculo-skeletal system in space and on earth. Eur. J. Appl. Physiol..

[8] Zhu H., Wang H., Liu Z. (2015). Effects of real and simulated weightlessness on the cardiac and peripheral vascular functions of humans: A review. Int. J. Occup. Med. Environ. Health.

[9] Graveline D.E. (1964). Cardiovascular deconditioning: Role of blood volume and sympathetic neurohormones. Life Sci. Space Res..

[10] Coupe M., Fortrat J.O., Larina I., Gauquelin-Koch G., Gharib C., Custaud M.A. (2009). Cardiovascular deconditioning: From autonomic nervous system to microvascular dysfunctions. Respir. Physiol. Neurobiol..

[11] Delp M.D., Charvat J.M., Limoli C.L., Globus R.K., Ghosh P. (2016). Apollo lunar astronauts show higher cardiovascular disease mortality: Possible deep space radiation effects on the vascular endothelium. Sci. Rep..

[12] Cucinotta F.A., Hamada N., Little M.P. (2016). No evidence for an increase in circulatory disease mortality in astronauts following space radiation exposures. Life Sci. Space Res..

[13] Rizzo A.M., Corsetto P.A., Montorfano G., Milani S., Zava S., Tavella S., Cancedda R., Berra B. (2012). Effects of long-term space flight on erythrocytes and oxidative stress of rodents. PLoS ONE.

[14] Guan J., Wan X.S., Zhou Z., Ware J., Donahue J.J., Biaglow J.E., Kennedy A.R. (2004). Effects of dietary supplements on space radiation-induced oxidative stress in Sprague-Dawley rats. Radiat. Res..

[15] Versari S., Longinotti G., Barenghi L., Maier J.A., Bradamante S. (2013). The challenging environment on board the International Space Station affects endothelial cell function by triggering oxidative stress through thioredoxin interacting protein overexpression: The ESA-SPHINX experiment. FASEB J..

[16] Mao X.W., Pecaut M.J., Stodieck L.S., Ferguson V.L., Bateman T.A., Bouxsein M., Jones T.A., Moldovan M., Cunningham C.E., Chieu J. (2013). Spaceflight environment induces mitochondrial oxidative damage in ocular tissue. Radiat. Res..

[17] Mao X.W., Pecaut M.J., Stodieck L.S., Ferguson V.L., Bateman T.A., Bouxsein M.L., Gridley D.S. (2014). Biological and metabolic response in STS-135 space-flown mouse skin. Free Radic. Res..

[18] Mao X.W., Nishiyama N.C., Pecaut M.J., Campbell-Beachler M., Gifford P., Haynes K.E., Becronis C., Gridley D.S. (2016). Simulated microgravity and low-dose/low-dose-rate radiation induces oxidative damage in the mouse brain. Radiat. Res..

[19] Wise K.C., Manna S.K., Yamauchi K., Ramesh V., Wilson B.L., Thomas R.L., Sarkar S., Kulkarni A.D., Pellis N.R., Ramesh G.T. (2005). Activation of nuclear transcription factor-κB in mouse brain induced by a simulated microgravity environment. In Vitro Cell Dev. Biol. Anim..

[20] Wang J., Zhang J., Bai S., Wang G., Mu L., Sun B., Wang D., Kong Q., Liu Y., Yao X. (2009). Simulated microgravity promotes cellular senescence via oxidant stress in rat PC12 cells. Neurochem. Int..

[21] Hollander J., Gore M., Fiebig R., Mazzeo R., Ohishi S., Ohno H., Ji L.L. (1998). Spaceflight downregulates antioxidant defense systems in rat liver. Free Radic. Biol. Med..

[22] Baqai F.P., Gridley D.S., Slater J.M., Luo-Owen X., Stodieck L.S., Ferguson V., Chapes S.K., Pecaut M.J. (2009). Effects of spaceflight on innate immune function and antioxidant gene expression. J. Appl. Physiol..

[23] Ikemoto M., Nikawa T., Kano M., Hirasaka K., Kitano T., Watanabe C., Tanaka R., Yamamoto T., Kamada M., Kishi K. (2002). Cysteine supplementation prevents unweighting-induced ubiquitination in association with redox regulation in rat skeletal muscle. Biol. Chem..

[24] Lawler J.M., Song W., Demaree S.R. (2003). Hindlimb unloading increases oxidative stress and disrupts antioxidant capacity in skeletal muscle. Free Radic. Biol. Med..

[25] Finkel T., Holbrook N.J. (2000). Oxidants, oxidative stress and the biology of ageing. Nature.

[26] Hayyan M., Hashim M.A., AlNashef I.M. (2016). Superoxide ion: Generation and chemical implications. Chem. Rev..

[27] Glasauer A., Chandel N.S. (2013). ROS. Curr. Biol..

[28] Valko M., Leibfritz D., Moncol J., Cronin M.T., Mazur M., Telser J. (2007). Free radicals and antioxidants in normal physiological functions and human disease. Int. J. Biochem. Cell. Biol..

[29] Mates J.M., Segura J.A., Alonso F.J., Marquez J. (2008). Intracellular redox status and oxidative stress: Implications for cell proliferation, apoptosis, and carcinogenesis. Arch. Toxicol..

[30] Kamata H., Hirata H. (1999). Redox regulation of cellular signalling. Cell. Signal..

[31] Sugamura K., Keaney J.F. (2011). Reactive oxygen species in cardiovascular disease. Free Radic. Biol. Med..

[32] Lambeth J.D. (2007). Nox enzymes, ROS, and chronic disease: An example of antagonistic pleiotropy. Free Radic. Biol. Med..

[33] Prieto-Bermejo R., Hernández-Hernández A. (2017). The Importance of NADPH oxidases and redox signaling in angiogenesis. Antioxidants.

[34] Gavazzi G., Banfi B., Deffert C., Fiette L., Schappi M., Herrmann F., Krause K.H. (2006). Decreased blood pressure in NOX1-deficient mice. FEBS Lett..

[35] Yetik-Anacak G., Catravas J.D. (2006). Nitric oxide and the endothelium: History and impact on cardiovascular disease. Vasc. Pharmacol..

[36] Li Q., Youn J.Y., Cai H. (2015). Mechanisms and consequences of endothelial nitric oxide synthase dysfunction in hypertension. J. Hypertens..

[37] Rochette L., Lorin J., Zeller M., Guilland J.C., Lorgis L., Cottin Y., Vergely C. (2013). Nitric oxide synthase inhibition and oxidative stress in cardiovascular diseases: Possible therapeutic targets?. Pharmacol. Ther..

[38] Sihver L., Ploc O., Puchalska M., Ambrozova I., Kubancak J., Kyselova D., Shurshakov V. (2015). Radiation environment at aviation altitudes and in space. Radiat. Prot. Dosim..

[39] Cucinotta F.A., Durante M. (2006). Cancer risk from exposure to galactic cosmic rays: Implications for space exploration by human beings. Lancet Oncol..

[40] Kryston T.B., Georgiev A.B., Pissis P., Georgakilas A.G. (2011). Role of oxidative stress and DNA damage in human carcinogenesis. Mutat. Res..

[41] Wang Y., Boerma M., Zhou D. (2016). Ionizing radiation-induced endothelial cell senescence and cardiovascular diseases. Radiat. Res..

[42] LaVerne J.A. (2000). Track effects of heavy ions in liquid water. Radiat. Res..

[43] Gonon G., Groetz J.E., de Toledo S.M., Howell R.W., Fromm M., Azzam E.I. (2013). Nontargeted stressful effects in normal human fibroblast cultures exposed to low fluences of high charge, high energy (HZE) particles: Kinetics of biologic responses and significance of secondary radiations. Radiat. Res..

[44] Li M., Gonon G., Buonanno M., Autsavapromporn N., de Toledo S.M., Pain D., Azzam E.I. (2014). Health risks of space exploration: Targeted and nontargeted oxidative injury by high-charge and high-energy particles. Antioxid. Redox Signal..

[45] Newberg A.B. (1994). Changes in the central nervous system and their clinical correlates during long-term spaceflight. Aviat. Space Environ. Med..

[46] DeFelipe J., Arellano J.I., Merchan-Perez A., Gonzalez-Albo M.C., Walton K., Llinas R. (2002). Spaceflight induces changes in the synaptic circuitry of the postnatal developing neocortex. Cereb. Cortex.

[47] Van Ombergen A., Demertzi A., Tomilovskaya E., Jeurissen B., Sijbers J., Kozlovskaya I.B., Parizel P.M., van de Heyning P.H., Sunaert S., Laureys S. (2017). The effect of spaceflight and microgravity on the human brain. J. Neurol..

[48] Kim J.S., Yang M., Kim S.H., Shin T., Moon C. (2013). Neurobiological toxicity of radiation in hippocampal cells. Histol. Histopathol..

[49] Gauger G.E., Tobias C.A., Yang T., Whitney M. (1986). The effect of space radiation of the nervous system. Adv. Space Res..

[50] Darby S.C., Ewertz M., McGale P., Bennet A.M., Blom-Goldman U., Bronnum D., Correa C., Cutter D., Gagliardi G., Gigante B. (2013). Risk of ischemic heart disease in women after radiotherapy for breast cancer. N. Engl. J. Med..

[51] Lipshultz S.E., Cochran T.R., Franco V.I., Miller T.L. (2013). Treatment-related cardiotoxicity in survivors of childhood cancer. Nat. Rev. Clin. Oncol..

[52] Boerma M., Nelson G.A., Sridharan V., Mao X.W., Koturbash I., Hauer-Jensen M. (2015). Space radiation and cardiovascular disease risk. World J. Cardiol..

[53] Soucy K.G., Lim H.K., Kim J.H., Oh Y., Attarzadeh D.O., Sevinc B., Kuo M.M., Shoukas A.A., Vazquez M.E., Berkowitz D.E. (2011). HZE ^56^Fe-ion irradiation induces endothelial dysfunction in rat aorta: Role of xanthine oxidase. Radiat. Res..

[54] Datta K., Suman S., Kallakury B.V., Fornace A.J. (2012). Exposure to heavy ion radiation induces persistent oxidative stress in mouse intestine. PLoS ONE.

[55] Qu L., Chen H., Liu X., Bi L., Xiong J., Mao Z., Li Y. (2010). Protective effects of flavonoids against oxidative stress induced by simulated microgravity in SH-SY5Y cells. Neurochem. Res..

[56] Gambara G., Salanova M., Ciciliot S., Furlan S., Gutsmann M., Schiffl G., Ungethuem U., Volpe P., Gunga H.C., Blottner D. (2017). Gene expression profiling in slow-type calf soleus muscle of 30 days space-flown mice. PLoS ONE.

[57] Salanova M., Gambara G., Moriggi M., Vasso M., Ungethuem U., Belavy D.L., Felsenberg D., Cerretelli P., Gelfi C., Blottner D. (2015). Vibration mechanosignals superimposed to resistive exercise result in baseline skeletal muscle transcriptome profiles following chronic disuse in bed rest. Sci. Rep..

[58] Wang Y., Javed I., Liu Y., Lu S., Peng G., Zhang Y., Qing H., Deng Y. (2016). Effect of prolonged simulated microgravity on metabolic proteins in rat hippocampus: Steps toward safe space travel. J. Proteome Res..

[59] Zhang R., Bai Y.G., Lin L.J., Bao J.X., Zhang Y.Y., Tang H., Cheng J.H., Jia G.L., Ren X.L., Ma J. (2009). Blockade of AT1 receptor partially restores vasoreactivity, NOS expression, and superoxide levels in cerebral and carotid arteries of hindlimb unweighting rats. J. Appl. Physiol..

[60] Zhang R., Ran H.H., Peng L., Xu F., Sun J.F., Zhang L.N., Fan Y.Y., Peng L., Cui G. (2014). Mitochondrial regulation of NADPH oxidase in hindlimb unweighting rat cerebral arteries. PLoS ONE.

[61] Peng L., Ran H.H., Zhang Y., Zhao Y., Fan Y.Y., Peng L., Zhang R., Cao F. (2015). NADPH Oxidase Accounts for Changes in Cerebrovascular Redox Status in Hindlimb Unweighting Rats. Biomed. Environ. Sci..

[62] Ran F., An L., Fan Y., Hang H., Wang S. (2016). Simulated microgravity potentiates generation of reactive oxygen species in cells. Biophys. Rep..

[63] Dikalov S. (2011). Cross talk between mitochondria and NADPH oxidases. Free Radic. Biol. Med..

[64] Stein T.P. (2002). Space flight and oxidative stress. Nutrition.

[65] Stein T.P., Leskiw M.J. (2000). Oxidant damage during and after spaceflight. Am. J. Physiol. Endocrinol. Metab..

[66] Da Silva M.S., Zimmerman P.M., Meguid M.M., Nandi J., Ohinata K., Xu Y., Chen C., Tada T., Inui A. (2002). Anorexia in space and possible etiologies: An overview. Nutrition.

[67] Heer M., Boerger A., Kamps N., Mika C., Korr C., Drummer C. (2000). Nutrient supply during recent European missions. Pflügers Arch. Eur. J. Physiol..

[68] Biolo G., Heer M., Narici M., Strollo F. (2003). Microgravity as a model of ageing. Curr. Opin. Clin. Nutr. Metab. Care.

[69] Adrian A., Schoppmann K., Sromicki J., Brungs S., von der Wiesche M., Hock B., Kolanus W., Hemmersbach R., Ullrich O. (2013). The oxidative burst reaction in mammalian cells depends on gravity. Cell. Commun. Signal..

[70] Veglia F., Cighetti G., de Franceschi M., Zingaro L., Boccotti L., Tremoli E., Cavalca V. (2006). Age- and gender-related oxidative status determined in healthy subjects by means of OXY-SCORE, a potential new comprehensive index. Biomarkers.

[71] Zhou Q., Li L., Zhao B., Guan K.L. (2015). The hippo pathway in heart development, regeneration, and diseases. Circ. Res..

[72] Porazinski S., Wang H., Asaoka Y., Behrndt M., Miyamoto T., Morita H., Hata S., Sasaki T., Krens S.F., Osada Y. (2015). YAP is essential for tissue tension to ensure vertebrate 3D body shape. Nature.

[73] Asaoka Y., Nishina H., Furutani-Seiki M. (2017). YAP is essential for 3D organogenesis withstanding gravity. Dev. Growth Differ..

[74] Vernikos J., Schneider V.S. (2010). Space, gravity and the physiology of aging: Parallel or convergent disciplines? A mini-review. Gerontology.

[75] Hohn A., Weber D., Jung T., Ott C., Hugo M., Kochlik B., Kehm R., Konig J., Grune T., Castro J.P. (2017). Happily (n)ever after: Aging in the context of oxidative stress, proteostasis loss and cellular senescence. Redox Biol..

